# Supporting People With Dementia in the Community: Occupational Therapy Service and Reimbursement Innovations

**DOI:** 10.1093/geroni/igaa057.2762

**Published:** 2020-12-16

**Authors:** Michael Lepore, Erin Long, Richard Fortinsky

## Abstract

The wide range of services needed to support a safe and quality life among people living with dementia at home is growing and extends beyond the bounds of traditional reimbursement models. Within the context of a health care system that is not designed to reimburse for these types of services, federal grants from the Alzheimer’s Disease Programs Initiative (ADPI) funded by the Administration for Community Living (ACL) have supported the delivery of home- and community-based services (HCBS) for people with dementia and their care partners with a pragmatic emphasis on sustainability, such as establishing successful reimbursement pathways. Drawing lessons from ACL’s ADPI program and from the Health Resources & Services Administration Geriatric Workforce Enhancement Program and Geriatrics Academic Career Award program, this symposium examines opportunities and strategies for providing services to people living with dementia in the community and highlights occupational therapy as a valuable dementia care service that has potential for sustainable delivery and opportunities for professional expansion. Papers address needed workforce development for delivering HCBS to diverse populations living with dementia and examine occupational therapy roles in delivering HCBS to persons with dementia and their care partners. Additionally, papers examine the implementation and outcomes of evidence-based occupational therapy and interprofessional interventions for persons living with dementia in the community. Reimbursement mechanisms for occupational therapy services delivered to people with dementia in the community are described. Discussion addresses how these innovative interventions and reimbursement mechanisms align with the recent surge of National Institute on Aging funding for pragmatic trials.

